# Designing Pancake-Bonded Heterodimers for Scanning Probe Microscopy

**DOI:** 10.1021/acs.jpca.5c08063

**Published:** 2026-03-26

**Authors:** Adam Matěj, Miklos Kertesz

**Affiliations:** † Department of Chemistry and Institute of Soft Matter, 8368 Georgetown University, Washington, DC 20057, United States; ‡ Institute of Physics of the Czech Academy of Sciences, Prague 162 00, Czechia; § Department of Physical Chemistry, Faculty of Science, Palacký University Olomouc, Olomouc 779 00, Czechia

## Abstract

Computational modeling of heterodimers of mono- and diradical molecules with C_60_^–^ indicates that scanning probe microscopy could characterize multicenter covalent-like interactions, also known as pancake bonds, on surfaces. By functionalizing the SPM tip with C_60_ and creating its monoanionic state by applying the corresponding voltage, individual pancake-bonded heterodimers with various radical molecules can be formed and characterized. All three proposed radical/diradical molecules readily form pancake-bonded dimers with C_60_^–^, and their structural and energetic minima are easily discernible from the dispersion-bonded van der Waals heterodimers with C_60_. Two of the three newly designed heterodimers show intermolecular C···C distances that are shorter than the current shortest pancake bond. This exploratory work aims to stimulate new studies on the interactions of radical molecules by the use of SPM techniques.

## Graphical abstract

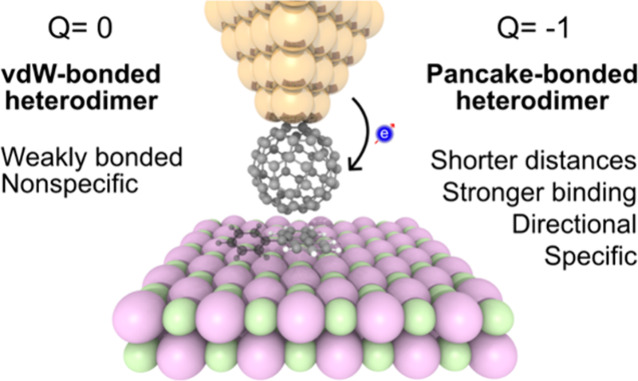

## Introduction

Conjugated planar organic molecules, aromatics, are well-known for their ability to form weakly π-bonded stacks. Generally, π-stacking is neither specific nor directional, as demonstrated by a flat potential energy surface of lateral displacement of closed-shell molecules.^1,2^ The picture changes once the molecules are open-shell.^3–5^ In certain cases, two or more interacting open-shell molecules can lower their total energy by forming multicenter electron-sharing covalent-like bonds, also known as pancake bonds.^6–11^ The pancake bond concept was first introduced in the 1960s by Mulliken,^6^ and since then, these complexes have been studied in the solid state^12–16^ and solution,^17–19^ with rare cases also in the gas phase.^20,21^ In applications of pancake-bonded complexes, one sees varied magnetism, highly conducting organic materials, and multifunctional aggregates. Pancake bonding interaction pairs the two frontier electrons over several atomic centers, usually driven by the overlap of the two delocalized π-stacked singly occupied molecular orbitals (SOMOs), one on each molecule, as illustrated in Figure 1A–C for homodimers on the prototypical phenalenyl dimer (PLY_2_). Pancake bonding is highly directional and specific, as any displacement that lowers the SOMO–SOMO overlap would result in a significant increase in energy.^22–24^ Besides stronger interaction than van der Waals (vdW), pancake bonding exhibits other characteristic properties, summarized in references.^10,25^

**Figure 1:**
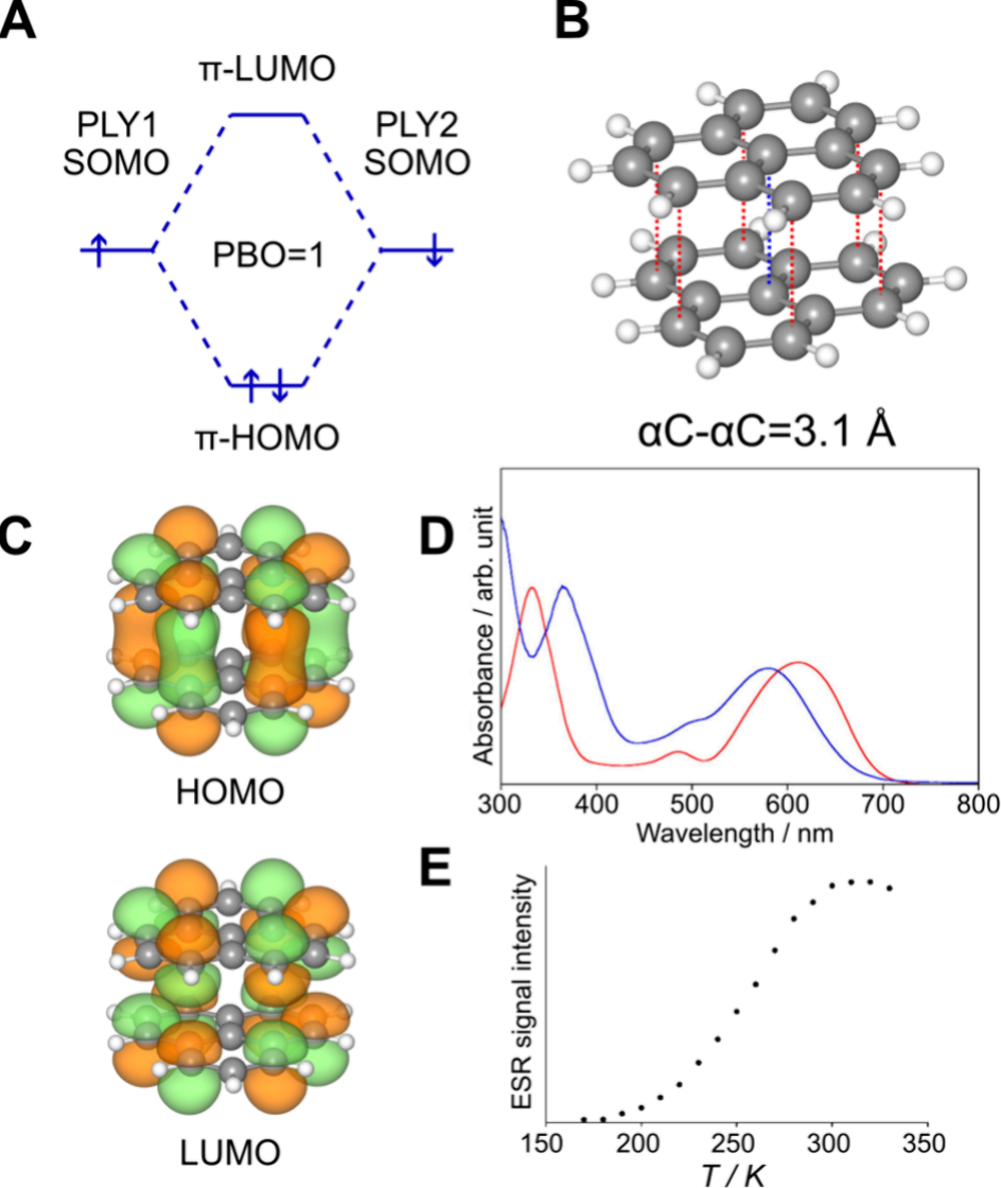
(A) MO diagram of phenalenyl π-dimer with pancake bond order of 1. (B) structure of pancake-bonded phenalenyl dimer, highlighting intermolecular distances between α-carbons in red. (C) frontier orbitals showcasing the nature of pancake bonding. (D) absorption spectra of triphenyl-phenalenyl (blue) and tBu-phenalenyl (red) π-dimers. (E) variable temperature ESR spectrum of triphenyl-phenalenyl dimer. Panels D and E are adapted (increased font size) with permission from ref 26. Copyright©2014 American Chemical Society.

In the PLY_2_ case, we can assign a formal pancake bond order (PBO) of 1, calculated by

$$\mathrm{PBO}=\frac{\sum \mathrm{bonding}\;\mathrm{electrons}-\sum \mathrm{antibonding}\;\mathrm{electrons}}{2}$$ 1

where the count is limited to those electrons participating in pancake bonding.^10^ In this paper, PBO = 1/2, corresponding to one shared electron, will also be considered. The lowest energy structure in the pancake-bonded PLY_2_ dimer shows a delocalized bonding MO, which is the highest occupied molecular orbital (HOMO) as a constructive combination of the two SOMOs, while the lowest unoccupied molecular orbital (LUMO) has intermolecular antibonding character (Figure 1C). In this geometry, the interplanar distance is 3.1 Å,^22^ well below the typical vdW value of 3.4 Å for carbon. This newly formed covalent-like bond results in a thermally accessible triplet state and a characteristic HOMO–LUMO optical transition in the visible red region (Figure 1D).^9,26^

Here, we propose suitable compounds for pancake-bonded heterodimers (hereafter referred to as dimers) assembled in situ using scanning probe microscopy (SPM). Due to its unprecedented resolution under low temperature and ultrahigh vacuum conditions, SPM has achieved groundbreaking measurements, including subatomic resolution of π- and σ-holes,^27,28^ elemental identification,^29,30^ tip-induced reactions,^31,32^ and individual spin excitations.^33–35^ For this reason, we envision that SPM will be capable of analyzing individual dimers from new angles, such as interaction profiles from atomic force microscopy force curves, radical center localization from 2D mapping of the interaction basin, and establishing novel single-molecule junctions involving radicals participating in pancake bonding. Should our proposed setup prove to be feasible, various radical molecules could be probed this way. In other words, we propose to use SPM to study pancake dimers from a new perspective, rather than improving STM or AFM itself. Nonetheless, unforeseen applications could arise from these experiments.

Within the context of this exploratory research, which tries to lay the groundwork for surface-assisted pancake bonds, the focus is solely on heterodimers formed between a functionalized SPM tip and an adsorbate. Importantly, by this approach, one can design pancake-bonded dimers by combining various radical molecules, thus studying the effects of SOMO–SOMO alignment and delocalization on the pancake bond strength. Particularly interesting could be the possibility of stabilizing the open-shell structures of diradicaloid molecules by forming pancake-bonded dimers. Furthermore, the newly formed dimers would be available for characterization by means of electronic transport measurements within scanning tunneling microscopy (STM) instrumentation, shedding light on their electronic structure and transport properties. Although STM operates in the tunneling-through-gap regime, it is employed in molecular junction transport measurements for its ability to manipulate single molecules.^36^ Finally, the precise control of the spatial arrangement between the two radical molecules would contribute to experimentally exploring via SPM the potential energy surface in real space around radicals and diradicals involved in pancake bonding.

The process of assembling these appropriate tip-adsorbate combinations requires on-surface synthesis (OSS)^37–39^ and tip functionalization techniques, as illustrated schematically in Figure 2.^40,41^ First, an open-shell molecule must be prepared on a surface (blue triangle in Figure 2), and since coplanarity of bonding atoms is required to form a successful pancake bond between π-MOs, these molecules should be nearly planar upon adsorption on the surface. Second, a suitable additional molecule must be attached to the SPM tip and capable of overlapping with the surface-adsorbed radical molecule. This requirement is strongly limiting, as attachment of polycyclic aromatic hydrocarbons to the SPM tip was shown to be preferably nonparallel with respect to the surface.^41,42^ For this reason, we propose to use C_60_ fullerene as the “pancake-sensing” molecule attached to the SPM tip (Figure 2, step 4). C_60_ molecules are routinely picked up with SPM tips,^43–45^ and their characteristic structure ensures that some portion of their carbon network will always be somewhat parallel to the underlying radical molecule of interest, as illustrated in Figure 2, point (6). In pancake bonding, one or two unpaired electrons are shared. Since neutral C_60_ is a closed-shell molecule, it would lead to a PBO = 1/2 when forming a pancake bond with a radical. On the other hand, the C_60_^–^ could, in principle, provide pancake bonding with a radical with PBO = 1 as in the case of PLY_2_ (Figure 1A). For this reason, both C_60_ and C_60_^–^ are explored as potential SPM-tip pancake bond sensors. Inducing charged states in individual molecules by applying bias voltage between the SPM tip and surface was shown to be a viable method to study the properties of charged species.^46^ By forming the charged species on an insulating layer, the discharge is effectively limited, which increases the lifetime of the ions.^47^ The successful formation of C_60_^–^ using SPM has been previously reported, and what is more, the voltage required for the formation of an anion can be significantly lowered by the combination of the work functions of the tip and surface.^48,49^ Charge density difference illustrating the accumulation of charge upon electron attachment on a gold tip-adsorbed C_60_ is shown in Supplementary Figure S1. To further justify the formation of C_60_^–^ by bias voltage applied between the tip and sample, the LUMO resonance energies from scanning tunneling spectroscopy experiments found in the original references are summarized herein. It was shown in ref 48 that on Au(111), the resonance of LUMO for adsorbed C_60_ is at 1 ± 0.2 V. For the molecules **1a**, **2**, and **3**, the respective LUMO resonances on Au(111) surface appear at 1.6, 1.2, and 0.5 V.^50–52^ The calculated electron affinities (see Supplementary Table S1) agree well with this trend, and it can be concluded that pancake-bonded dimers can be formed at bias voltage around 1 V for molecules **1a** and **2**, while possibly a lower voltage, between 0.5 and 1 V, could yield the desired dimers for **3**. Overall, the required voltages are well within the range of standard STM measurements.

**Figure 2:**
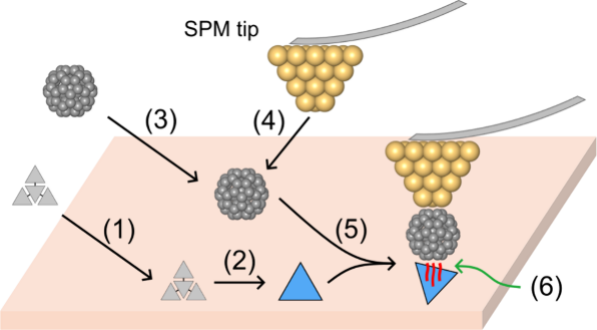
Scheme illustrating the principle of forming in situ pancake-bonded heterodimers using scanning probe microscopy. (1) Molecular precursors are deposited on the surface. (2) Precursors are transformed into the final open-shell molecules using OSS techniques. (3) Deposition of C_60_ on the surface. (4) Approach by the SPM tip to the C_60_ molecule, and subsequent attachment to the tip. (5) Approaching an individual (di)­radical molecule with a C_60_-decorated metallic tip leads to the formation of a dimer between the molecule on the surface and C_60_ on the tip. (6) The interaction within the formed dimer is the main subject of this work.

Standard density functional theory (DFT) methods were used to screen potentially suitable systems and further analyze their properties (for calculation details, see Methods). The selection of radical and diradical molecules (hereafter referred to as (di)­radicals) for the role of the surface adsorbed molecule (blue triangle in Figure 2) consists of three π-conjugated molecules. All three have been previously synthesized on clean metallic surfaces by OSS. Their structures and unpaired electron density plots in the planar forms (*vide infra*) are shown in Figure 3. The first and smallest is the phenyl-fluorenyl radical **1a**, which was reported and characterized on the Au(111) surface by Zhai et al.^50^ This molecule will be discussed, alongside its hydrogenated closed-shell derivative **1b**, in greater detail. Hydrogenation as a model here allows us to probe the role of the unpaired electrons in pancake bonding, as the single extra hydrogen positioned away from the unpaired electron density center introduces only a negligible perturbation of the vdW interaction while it removes the unpaired electron. The second molecule, **2**, is a diradical, observed on the Au(111) surface by Mishra et al.^51^ The last proposed molecule, **3**, was reported previously by Frezza et al. on the Au(111) surface.^52^ All three molecules show various degrees of nonplanarity in the gas phase (see Figure S2), but importantly, when adsorbed on the surfaces, they become nearly planar. Their plane-constrained geometries, denoted by a **/p** suffix, serve as simple models of surface-induced planarization. Details on the constraints are described in the Methods section.

**Figure 3:**
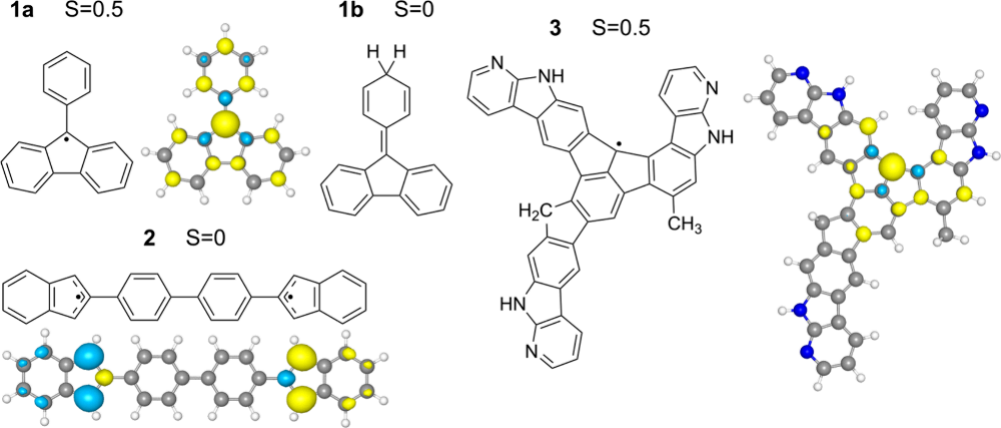
Structures of the proposed precursors **1a**, **1b**, **2**, and **3** are illustrated as blue triangles in Figure 2. Computed spin densities are obtained from spin-unrestricted DFT of the ground-state open-shell structures in their planarized constrained geometries **1a/p**, **2/p**, and **3/p**. Total spin, S, is included for each structure. Yellow and light blue isosurfaces represent α and β spin densities, respectively.

This article is organized as follows: first, we discuss dimers of these four molecules in their planarized constrained geometries **1a/p**, **1/b/p**, **2/p**, and **3/p**, combined with both neutral and anionic C_60_ as a model for surface-adsorbed planarized molecules. Then, all systems are relaxed on a periodic NaCl(001) surface to track any changes from the idealized planar model.

## Methods

All calculations in the gas phase were done using the quantum chemistry code ORCA version 6.1.0.^53^ The level of theory was chosen to be a global hybrid meta-GGA functional from the Minnesota family with double the amount of HF exchange, M06-2X;^54^ the basis set for geometry relaxations was def2-SVP, with def2-TZVP being used for single-point energy calculations.^55^ Resolution of identity approximation was used for Coulomb integrals, i.e., AuxJ basis.^56^ The respective in-plane optimized structures are referred to as **1a/p**, **1b/p**, **2/p**, and **3/p**. In the cases of constrained planar gas phase relaxations (**/p** suffix), the z Cartesian coordinates of the radical/diradical molecule were fixed, and relaxation was allowed only within the xy plane. For **1a/p**, **1b/p,** and **3/p**, some atoms were allowed to relax fully due to high steric hindrance or sp^3^ hybridization (see Figure S3). All cutoffs were kept default in accordance with TightSCF and DEFGRID3 settings. Frequency calculations were performed in each relevant structure to ensure that a minimum was reached. In the case of the open-shell singlet ground state (molecule **2** and **2/p**), the broken symmetry solution was targeted and checked for internal and external instabilities by setting options “StabPerform″ and “StabRestartUHFifUnstable″ to “true″ within the SCF block.

Calculations with a periodic surface were done in the program FHI-AIMS, version 250320_1.^57^ The NaCl(001) monolayer was modeled as a 5 × 5 × 1 square slab of 200 atoms using the lattice constant of 5.620 Å. The atomic positions of the slab were frozen throughout the relaxation steps. The supercell had final dimensions 28.10 × 28.10 × 100 Å to ensure a large enough vacuum gap between images in the z direction. For geometry optimization, the standard GGA PBE functional was used.^58^ After convergence on an initial light basis tier-1, the structures were further relaxed on a tight tier-2 basis. The dispersion correction by Tkatchenko and Scheffler^59^ was employed for the relaxation steps. After convergence, single-point calculations with the M06-2X functional at a tight tier-2 basis without additional dispersion correction were done. Due to errors introduced by jellium in charged supercells, nonperiodic single-point energy calculations were carried out on fixed structures. Energies and MOs are presented from these “cluster″ calculations, while Mulliken atomic charges are from the periodic calculations. Mulliken charges show negligible variation between periodic and cluster calculations. For all calculations, the scalar-relativistic approximation termed atomic-ZORA^57^ was turned on by default. For the periodic surface, a 2 × 2 × 1 Gamma-centered *k*-mesh was used. Convergence cutoffs for SCF cycles were set to 10^–5^ e/a_0_^3^ for electron density and 10^–4^ eV for total energy. The minimum absolute force component for the BFGS geometry optimization was set to 5·10^–3^ eV/Å. ZPVE corrections were not calculated for any structures in the FHI-AIMS program.

Per-fragment charges, ∑*q*, were calculated as a sum of individual Mulliken atomic charges through atoms on each fragment. Visual Molecular Dynamics (VMD) version 1.9.4a57 was used for the images in the figures.^60^

Molecular substitutions discussed in Figure 4 were done as follows. The relaxed **1b/p** molecule was inserted in the place of **1a/p** within the **1a/p**–C_60_ dimer (Figure 4C) and the **1a/p**–C_60_^–^ dimer (Figure 4E-F). The alignment of **1b/p** was done by minimizing the root mean squared deviation (RMSD) of the five carbon atoms forming the 5-membered ring of the fluorenyl moiety. VMD program was used for this alignment. These aligned structures, illustrated in Figure 4C,E,F, are shown in more detail in Supplementary Figure S4 together with their RMSD values.

**Figure 4:**
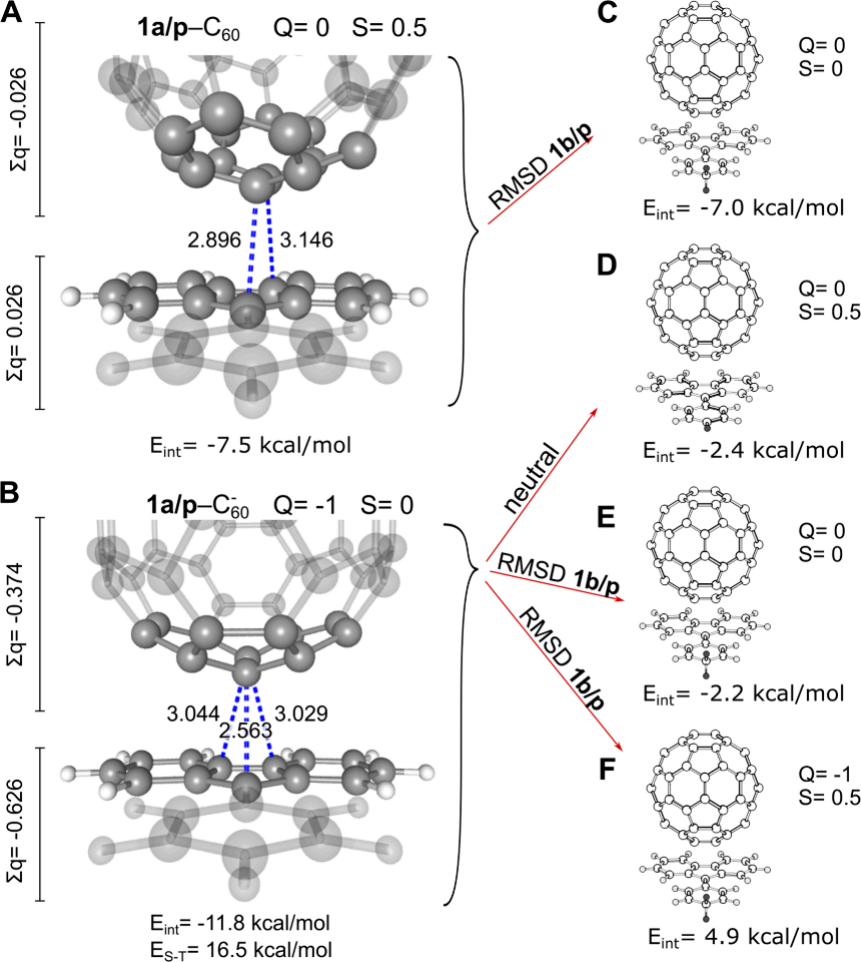
Relaxed minimum structures of **1a/p**–C_60_ (A) and **1a/p**–C_60_^–^ (B). Sums of Mulliken atomic charges (∑*q*) are on the margins of respective fragments. Each panel contains the total charge *Q* and total spin *S* of the dimer. Panels (C, E, F) illustrate single-point calculations in various charge and multiplicity states at fixed geometries obtained via substitution (by minimizing RMSD fit as defined in the Methods section) of **1a/p** by **1b/p**. (C) **1b/p**–C_60_ with **1b/p** substituted into **1a/p**–C_60_; (D) **1a/p**–C_60_ at geometry of **1a/p**–C_60_^–^; (E) **1b/p**–C_60_ with **1b/p** substituted into **1a/p**–C_60_^–^; (F) **1b/p**–C_60_^–^ with **1b/p** substituted into **1a/p**–C_60_^–^. All distances are in Å.

## Results and Discussion

Initially, we introduce the interaction of C_60_ with the three selected (di)­radical molecules serving to model surface-adsorbed molecules. Imposing planarity on these molecules allows us to controllably examine the propensity toward forming pancake bonds with C_60_ and C_60_^–^. Molecules **1a/p** and **1b/p** were used for the analysis of various types of bonding. Figure 4 shows the structural and energetic characteristics of the minima of **1a/p**–C_60_ and **1a/p**–C_60_^–^. In Figure 4A, the interaction energy of **1a/p–**C_60_ reaches −7.5 kcal/mol along with negligible charge transfer, denoted as ∑*q* (for details see the Methods section). The in-plane constrained optimized geometry exhibits multiple short carbon–carbon contact distances between the two fragments that are shorter than the sum of the vdW radii. In order to establish the amount of interaction due to the unpaired electron, **1a/p** was replaced with the relaxed structure of **1b/p** (for alignment of structures, see Methods section), and the single-point energy of this closed-shell neutral system was calculated; this is illustrated in Figure 4C. In this case, the interaction energy is −7.0 kcal/mol, only 0.5 kcal/mol different from that of **1a/p**–C_60_. This negligible difference clearly indicates that the radical **1a/p**–C_60_, a dimer formally able to form a pancake bond with PBO = 1/2, and the closed shell **1b/p**–C_60_, PBO = 0, both interact only via vdW interaction, without any pancake bond stabilization. The sub-vdW distances can be rationalized in this case by the large number of attractive vdW C···C interactions in the vicinity of close contacts. As a result, the cumulative attractive interactions from multiple carbon atoms in the vicinity of the close contact region outweigh the repulsions from the few close contacts. Interesting to note in this context that the single crystal XRD structure of C_60_ contains a large number of short C···C contacts below 3.14 Å,^61^ likely for the same reason.

In the case of **1a/p**–C_60_^–^, a significant increase in the interaction energy and a shortening of the intermolecular distances can be seen, as illustrated in Figure 4B. The interaction energy of −11.8 kcal/mol, together with the shortest intermolecular C···C distance of 2.563 Å, is a strong indication of a pancake bond with PBO = 1. The ∑*q* metric shows a significant charge transfer from the C_60_^–^. By comparing the singlet and triplet energy splitting (at singlet geometry), it was previously shown that the difference can be approximately attributed to the SOMO–SOMO bonding combination.^62^ Accordingly *E*_SOMO–SOMO_^int^, the driver of the pancake bond can be approximated by the singlet–triplet energy splitting, *E*_S–T_, and the total interaction energy in a pancake-bonded system by the sum of a vdW term and the SOMO–SOMO term:

$$E_{P.C.}^{\mathrm{int}}\approx E_{\mathrm{vdW}}^{\mathrm{int}}+E_{\mathrm{SOMO}-\mathrm{SOMO}}^{\mathrm{int}}$$ 2

$$E_{\mathrm{SOMO}-\mathrm{SOMO}}^{\mathrm{int}}\approx E_{S-T}=E_{S}-E_{T}$$ 3

Here, the calculated *E*_S–T_ = −16.5 kcal/mol, leading to an estimated *E*_vdW_^int^ ≈ −11.8 + 16.5 = 4.7 kcal/mol, a positive value reasonably corresponding to a repulsive vdW interaction given the short intermolecular contacts in the optimized **1a/p**–C_60_^–^ dimer. Similarly, to the model dimer in Figure 4C, three other model dimer geometries were investigated, as shown in panels D–F. Figure 4D illustrates **1a/p**–C_60_ at the fixed **1a/p**–C_60_^–^ geometry. The difference in interaction energy comes solely from removing the extra electron, thus making the dimer globally a neutral doublet. The interaction of only −2.4 kcal/mol, compared to −11.8 kcal/mol, by simply removing the extra electron, highlights the importance of creating C_60_^–^ in this study. In Figure 4E, **1b** **/p** was put in place of **1a/p** in the **1a/p**–C_60_^–^ dimer and calculated as the neutral singlet, thus changing PBO = 1 to 0. The interaction energy is −2.2 kcal/mol, very close to −2.4 kcal/mol for the case of **1a/p**–C_60_ at the anionic dimer geometry (Figure 4D). We attribute this weak attraction to the deformation energy of C_60_ at C_60_^–^ geometry, as these PBO = 1/2 and 0 should show a repulsive interaction, as demonstrated by the calculated *E*_S–T_ above. It is imperative to mention that this interaction is much weaker than the −7.5 kcal/mol obtained for vdW-bonded **1a/p**–C_60_, confirming pure vdW interaction. Lastly, the **1b/p** was substituted into the **1a/p**–C_60_^–^ dimer geometry (Figure 4F), globally anionic doublet with possible PBO = 1/2, which results in interaction energy +4.9 kcal/mol. This repulsion agrees strikingly well with the estimation of *E*_vdW_^int^ ≈ +4.7 kcal/mol calculated by eqs 2 and 3. These results show that the pancake-bonded dimer **1a/p**–C_60_^–^ is a truly unique local minimum not present in any other combination of the two molecules. By performing this comparison, it was shown that the C_60_ is not able to form pancake-bonded dimers with PBO lower than 1 as they do not result in stabilization beyond the vdW interactions.

The last, and arguably the most illustrative characteristic of the pancake bond is the bonding molecular orbital, which by definition must show overlap between multiple centers. Figure 5A shows the bonding HOMO of **1a/p**–C_60_^–^, highlighting overlap between a total of five carbon atoms: three from **1a/p** and two from C_60_^–^. With all this evidence, it can be concluded that this anionic dimer forms a two-electron/five-center (2e^–^/5c) pancake bond with PBO = 1.

**Figure 5:**
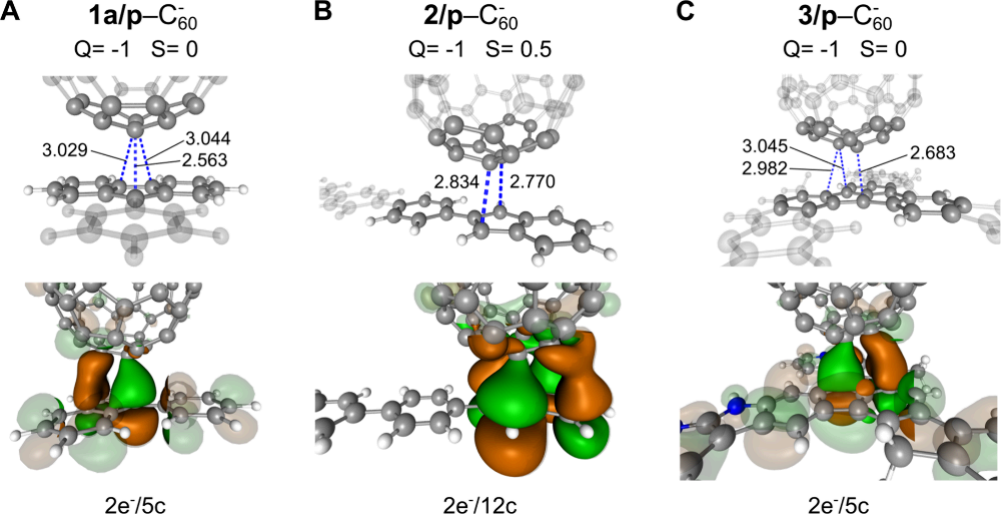
Structures (top row) and bonding orbitals (bottom row) of pancake-bonded dimers. Close contacts are highlighted by dashed blue lines with reported values in Å. The most important portions of the MOs are drawn in opaque colors, while the surrounding parts of the MOs are rendered translucent for clarity. (A) **1a/p**–C_60_^–^, globally singlet. (B) **2/p**–C_60_^–^, globally doublet. (C) **3/p**–C_60_^–^, globally singlet.

After analyzing that C_60_^–^ is truly necessary and can in fact form pancake bonds with radical molecules, we move on to discuss the results of the rest of the proposed molecules. Figure 5B,C shows the computed minimal energy structures of C_60_^–^ with **2/p** and **3/p**, respectively. All dimers exhibit imaginary frequencies for a very limited number of out-of-plane vibrations, which stem from the planarity constraints and do not contain significant normal mode components near the pancake-bonded region. Table 1 summarizes the interaction energies obtained by eqs 4 and 5:

$$E_{\mathrm{int}}=E_{\dim\mathrm{er}}^{Q}-E_{\mathrm{mol}}^{0}-E_{C_{60}^{Q}}^{Q}$$ 4

$$E_{\mathrm{int}+\mathrm{ZPVE}}=E_{\mathrm{int}}+{\mathrm{ZPVE}}_{\dim\mathrm{er}}-{\mathrm{ZPVE}}_{\mathrm{mol}}-{\mathrm{ZPVE}}_{C_{60}}$$ 5

**Table 1:** Interaction Energies and Summed Atomic Mulliken Charges (∑*q*) of Plane-Constrained Dimers^a^ in Two States, Neutral and Anionic^b^

	neutral (*Q* = 0)			Anion (Q = −1)		
M06-2X def2-TZVP	*E*_int_ [kcal/mol]	∑*q*_mol_	∑*q*_C_60_^Q^_	*E*_int_ [kcal/mol]	∑*q*_mol_	∑*q*_C_60_^Q^_
**1a/p**–C_60_^Q^	–8.1 (−7.5)	0.026	–0.026	–15.3 (−11.8)	–0.626	–0.374
**1b/p**–C_60_^Q^	–7.9 (−7.2)	0.032	–0.032	–5.2 (−4.2)	–0.030	–0.970
**2/p**–C_60_^Q^	–7.3 (−6.8)	0.026	–0.026	–14.7 (−11.1)	–0.717	–0.283
**3/p**–C_60_^Q^	–9.7 (−9.1)	0.028	–0.028	–18.1 (−17.5)	–0.774	–0.226

a Constrained geometry optimization is detailed in the Methods section.

b Values in brackets include ZPVE corrections.

By analyzing the neutral and anionic states along with their bonding orbitals, it is evident that all three (di)­radical molecules form stable pancake-bonded dimers of PBO = 1 with C_60_^–^. In cases **2/p**–C_60_^–^ and **3/p**–C_60_^–^, the pancake bonds are described as 2e^–^/12c and 2e^–^/5c bonds, respectively. Supplementary Figure S5 collects the stable minima of neutral dimers **1a/p**–C_60_, **1b/p**–C_60_, **2/p**–C_60_, and **3/p**–C_60_ together with their calculated spin densities. For comparison, the unconstrained structures, their calculated spin densities, and the bonding orbitals are in Figures S6 and S7. From the structures, bonding MOs, and interaction energies (Supplementary Table S2), it is clear that the observed pancake-bonded minima are not artifacts due to induced planarization. The small differences in interaction energies between planarized (/p) and unconstrained dimers in the gas phase further indicate that out-of-plane vibrations with imaginary frequencies do not affect bonding.

The competition between σ-bonding and pancake bonding is known in the literature,^9,26,63^ and it deserves a brief mention here despite being out of the scope of this work. In addition to the observed pancake bonds, we observed one case of a σ-bonded **1a**–C_60_^–^ dimer during computational modeling in gas phase. The largest difference between σ- and pancake-bond is in the hybridization. While the pancake-bonded dimers retain planarity, the carbons forming a covalent σ bond follow sp^2^–sp^3^ hybridization, resulting in a nonplanar structure. Since in the case of **1a,** the radical center resides on the apical carbon of fluorenyl, the out-of-plane distortion includes the whole phenyl moiety (Figure S8). This covalency yields a rather long and weak C–C bond of 1.605 Å and an interaction energy of −17.9 kcal/mol. We want to stress that this distortion from planarity is prohibited by the presence of the surface, and thus, only dispersion and pancake interactions are expected to be present in the proposed setup.

Finally, after establishing that the proposed systems can form pancake bonds under certain conditions, the dimers were relaxed on a fixed periodic monolayer of NaCl terminated in the [001] direction. This approach was chosen to model the common experimental conditions for charged species. Due to the requirement for a charged species, it is expected that the measurements will be carried out on a NaCl insulator layer to decouple the anion from the metallic substrate and thus slow down the discharge of anions.^41,64,65^ The metallic substrate will most likely be Au(111), which is required for the OSS reactions.

The interaction energy on the surface was calculated according to eq 6:

$$E_{\mathrm{int}}=E_{\dim\mathrm{er}@\mathrm{surf}}^{Q}-E_{\mathrm{mol}@\mathrm{surf}}^{0}-E_{C_{60}^{Q}}^{Q}$$ 6

where *E*_dimer@surf_^Q^ is the total energy of the adsorbed dimer with the surface, *E*_mol@surf_^0^ is the total energy of the adsorbed (di)­radical molecule with the surface, ${E_{{C_{60}}^{Q}}}^{Q}$ is the total energy of C_60_^Q^ in the vacuum. Superscripts denote which terms were calculated at the dimer’s total charge and which as neutral. First, the (di)­radical molecules (**1a**, **2**, and **3**) were relaxed on the NaCl(001) surface, and the total energies were taken as the *E*_mol@surf_^0^ references for eq 6. After this initial relaxation, C_60_ and C_60_^–^ were placed into positions based on the gas-phase results. The results shown in Figure 6 confirm the successful formation of the pancake-bonded dimers of PBO = 1. Due to an error in the total energies, introduced by jellium background charge, the interaction energies were calculated from single-point cluster calculations (see Methods and Supplementary Discusion for details). All three systems are found to interact via weak vdW interactions with C_60_ (see Figure S9 for spin densities and Table 2), while the C_60_^–^ readily forms pancake bonds with the proposed open-shell molecules.

**Table 2:** Interaction Energies and Summed Atomic Mulliken Charges (∑*q*) of Surface-Adsorbed Dimers in Two States, Neutral and Anionic, for Periodic Models Including the NaCl Monolayer As Shown in Figure 6 ^a^

	neutral (*Q* = 0)				anion (*Q* = −1)			
M06-2X tight-tier 2	*E*_int_ [kcal/mol]	∑*q*_mol_	∑*q*_C_60_^Q^_	∑*q*_surf_	*E*_int_ [kcal/mol]	∑*q*_mol_	∑*q*_C_60_^Q^_	∑*q*_surf_
**1a**–C_60_^Q^	–8.9	0.105	–0.094	–0.011	–20.1	–0.528	–0.368	–0.105
**2**–C_60_^Q^	–6.5	0.090	–0.070	–0.019	–22.8	–0.564	–0.313	–0.123
**3**–C_60_^Q^	–6.2	0.106	–0.112	0.006	–22.3	–0.629	–0.271	–0.100

a Interaction energies were calculated according to eq 6. All values refer to fully optimized geometries.

**Figure 6:**
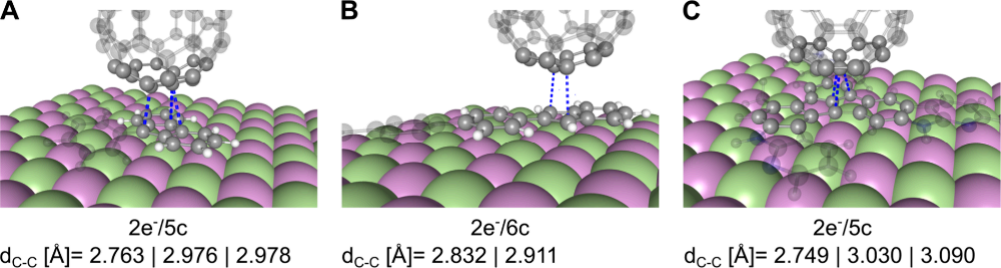
Anionic dimers relaxed on a fixed periodic NaCl(001) monolayer. (A) **1a**–C_60_^–^, (B) **2**–C_60_^–^, (C) **3**–C_60_^–^. Dashed blue lines highlight close contacts, and their values are listed below the images. Side structures are plotted translucent for clarity. Pancake bond characteristics are given below the structures. Mauve and lime balls represent Na and Cl, respectively.

Interaction energies for neutral dimers in Table 2 agree well with the in-plane constrained dimers summarized in Table 1. Despite all three dimers being formally able to form dimers with PBO = 1/2, they are found to interact only by vdW attraction. The relaxed structures of neutral vdW dimers are shown in Figure S9 together with their calculated spin densities. The relaxed dimers with C_60_^–^, shown in Figure 6, show qualitatively the same picture as with the plane-constrained (di)­radical molecules discussed in Figure 5. For the three studied open-shell molecules, **1a**, **2**, and **3**, their pancake bonding characteristics with C_60_^–^ (PBO = 1) are 2e^–^/5c, 2e^–^/12c, and 2e^–^/5c, respectively, and their bonding MOs are plotted in Figure S10. It can be seen that all three dimers adopt longer intermolecular distances on the surface, compared to their gas-phase models, without changing the shapes of the bonding MOs significantly. Thus, the plane-constrained (di)­radicals represent good models for surface-adsorbed pancake-bonded systems.

Regarding the negatively charged systems in Table 2, one can see a significant increase in the interaction energies compared to those without the NaCl surface (Tables 1 and supplementary Table S2). As a comparison, the interaction energies of the isolated dimers at fixed geometries as excised from the full optimized computation including the NaCl surface were calculated (see Table S3) using eq 5, and the values agree well with the results shown in Table 1. Here, the C_60_^Q^ molecule approaches the surface-adsorbed (di)­radical molecule, and the interaction energy corresponds to the partition presented by eq 6. As can be seen from the *E*_int_ values in Table 2, the interaction energy is significant, and in all cases, more than double that of neutral vdW dimers. Table 3 offers a comparison of interaction energies for gas-phase plane-constrained dimers and surface-adsorbed dimers. It is clearly seen from the interaction energies that gas-phase planarized molecules serve as great models for the surface-adsorbed vdW-bonded dimers with C_60_. More importantly, the pancake-bonded dimers with C_60_^–^ are further stabilized by the presence of NaCl surface, making the pancake-bonded minima significantly separated from the vdW-bonded dimers. With these energetically well-separated, distinct, and highly specific bonded minima, contrary to vdW interaction, we believe that the experiments would be able to unambiguously locate these pancake-bonded dimers.

**Table 3:** Comparison of Interaction Energies between Neutral and Charged Heterodimers in Plane-Constrained Gas-Phase Geometries (gp/p) and Surface-Adsorbed Geometries (surf)

	neutral (*Q* = 0)		anion (*Q* = −1)	
*E*_int_ [kcal/mol]	gp/p	surf	gp/p	surf
**1a**–C_60_^Q^	–8.1	–8.9	–15.3	–20.1
**2**–C_60_^Q^	–7.3	–6.5	–14.7	–22.8
**3**–C_60_^Q^	–9.7	–6.2	–18.1	–22.3

At this point, we want to acknowledge the extraordinarily short distances in pancake-bonded dimers recorded herein. To the best of our knowledge, the currently shortest pancake bond was measured at 2.80 Å by Piccoli et al.,^13^ making the heterodimers with **1a**–C_60_^–^ and **3**–C_60_^–^ contestants for the shortest C–C pancake bond.

To summarize the main points: all three presented modeling (isolated molecules with and without planarization and surface adsorbed modeling) consistently point toward pancake bonding with short distances and significant binding energies between the SPM tip (represented by C_60_^–^) and any of the three adsorbed molecules. This opens the possibility to experimentally test and explore this predicted phenomenon.

## Conclusions

We presented three open-shell π-conjugated organic molecules as possible candidates to form pancake bonds using advanced scanning probe microscopy experiments. All three molecules were previously reported on surfaces by means of on-surface synthesis under ultrahigh vacuum conditions within scanning probe microscopy instrumentation. Fullerene C_60_ was proposed as a functionalized tip for the formation of a pancake bond with the adsorbed radical molecules. We have shown that neutral C_60_ lacks a suitable electronic structure for pancake bonding and forms weak van der Waals bonds even with radicals in this study. Charging the fullerene molecule to its monoanionic state leads to the successful formation of pancake-bonded heterodimers, suitable for in situ measurements using scanning probe microscopy. By a stepwise analysis starting from constrained gas phase models moving to unconstrained surface dimers, we were able to analyze the geometry, strength, and character of these pancake bonds. Furthermore, the results suggest that two of the designed heterodimers could contest for the shortest recorded pancake bond to date. We hope that this theoretical investigation into suitable systems will stimulate experimental endeavors into further study of these partially covalent complexes.

## Supplementary Material



## Notes

Additional figures discussed in this article and comments on the use of cluster calculations; all the data supporting this study; all discussed structures, calculated spin densities, and molecular orbitals accessible from the data repository at 10.5281/zenodo.15795187 (PDF)
