# Supplementary material for: Designing Pancake-Bonded Heterodimers for Scanning Probe Microscopy
Source: J Phys Chem A. 2026 Mar 26;130(18):3525–33. doi: 10.1021/acs.jpca.5c08063 (PMC13158990; doi:10.1021/acs.jpca.5c08063)
Supplement: Supplementary file 1 [file jp5c08063_si_001.pdf]

Supplementary Information

Designing Pancake-Bonded Heterodimers for Scanning-Probe Microscopy

Adam Matěj[1,2,3,*], Miklos Kertesz[1]

1 Department of Chemistry and Institute of Soft Matter, Georgetown University, Washington, 20057, DC, USA

2 Institute of Physics of the Czech Academy of Sciences, Prague, 162 00, Czechia

3 Department of Physical Chemistry, Faculty of Science, Palacký University Olomouc, Olomouc, 779 00, Czechia

* Corresponding author: Adam Matěj, adam.matej@upol.cz

Table of contents

1. Fig S1: Charge distribution in tip-adsorbed C_{60}^{-}
2. Table S1: Calculated electron affinities
3. Fig S2: Nonplanarity of studied radical/diradical molecules
4. Fig S3: Definitions of planarization constraints
5. Fig S4: Fitting of **1b/p** over **1a/p** by minimizing RMSD
6. Fig S5: Spin densities of vdW-bonded dimers of planarized molecules with C_{60}
7. Fig S6: Structures and bonding MOs of unconstrained dimers with C_{60}^{-}
8. Fig S7: Spin densities of vdW-bonded dimers of unconstrained dimers with C_{60}
9. Table S2: Interaction energies and Mulliken charges of gas-phase unconstrained dimers
10. Fig S8: σ-bonded dimer of **1a**–C_{60}^{-}
11. Comments on cluster calculations
12. Fig S9: Spin densities of vdW-bonded dimers with C_{60} on periodic NaCl
13. Fig S10: Structures and bonding MOs of dimers with C_{60}^{-} on periodic NaCl
14. Table S3: Interaction energies and Mulliken charges of isolated dimers from the surface at fixed geometries

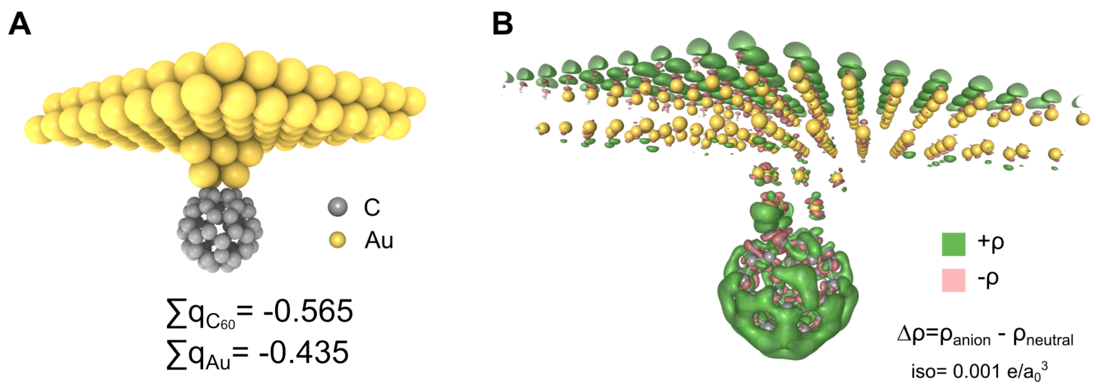

Figure S1: (A) structure of relaxed C_{60}^{-} on the gold tip with a periodic Au(111) slab. (B) Charge density difference isosurface, calculated as a difference between anion and neutral system at anionic geometry. Green and pink isosurfaces show charge accumulation and depletion after electron attachment, respectively.

Table S1: Electron affinities calculated at def-TZVP basis in planarized (/p) geometries in the gas phase. Values are in kcal/mol, calculated as the energy difference between the relaxed anion and neutral states.

gp /p	**1a**	**2**	**3**	$\mathbf{C_{60}}$
EA [kcal/mol]	50.4	52.7	64.6	58.2

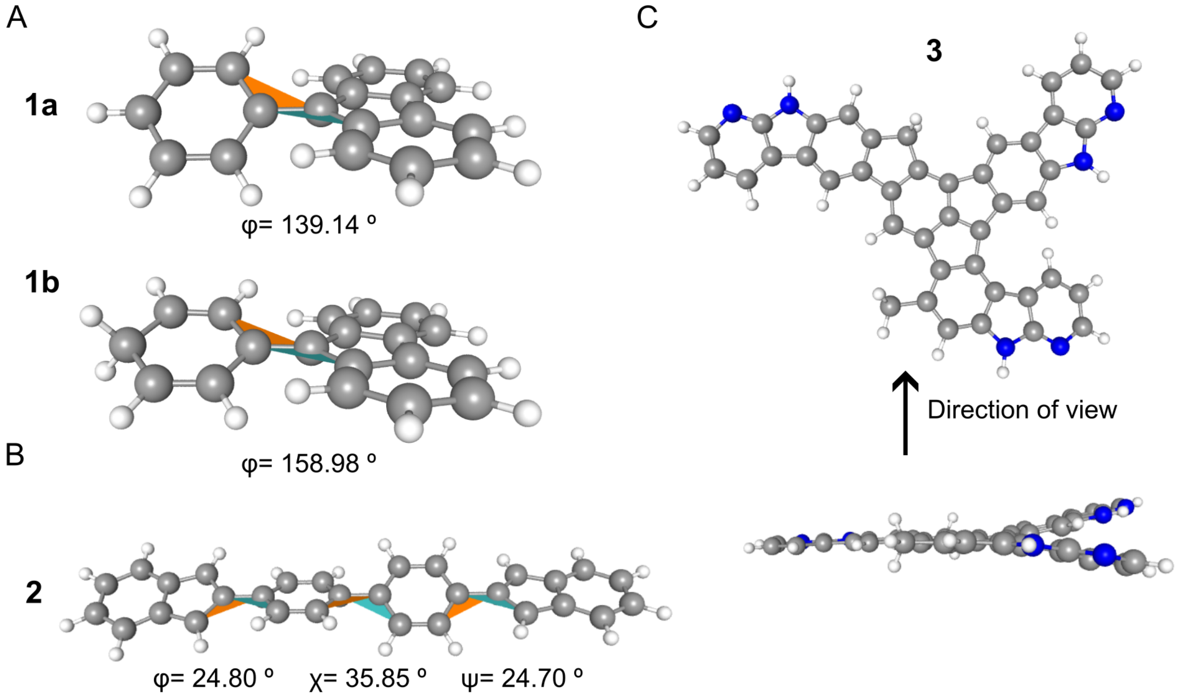

Figure S2: Relaxed unconstrained structures of molecules (a) **1a** and **1b**, (b) **2,** and (c) **3** in the gas phase. Dihedral angles for molecules **1a**, **1b**, and **2** are highlighted by cyan and orange planes. For molecule **3**, the side view is in the direction of the arrow, highlighting the out-of-plane distortion of the side chains.

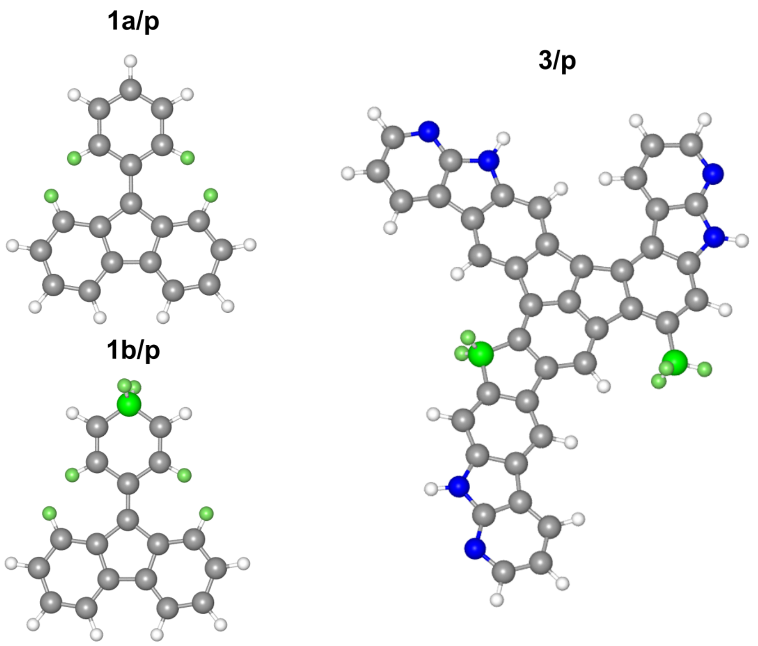

Figure S3: Atoms highlighted in green (C) and lime (H) were allowed to relax in all directions. Remaining atoms were fixed in the XY plane, including the entirety of molecule **2/p**.

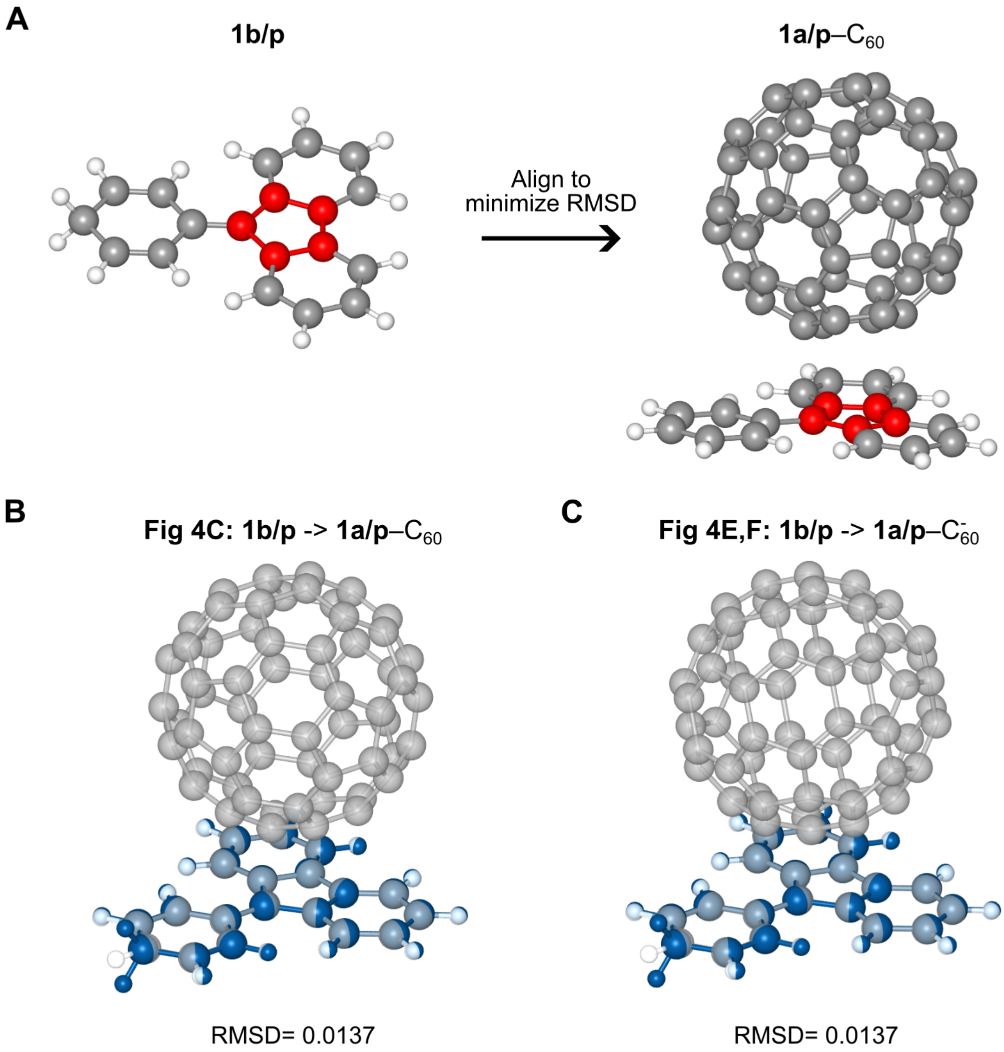

Figure S4: (A) illustration of alignment process. Root mean squared deviation (RMSD) of 5 highlighted atoms was minimized and the newly aligned **1b/p** was substituted in place of **1a/p** in the two dimers. Aligned **1a/p** (B) and **1b/p** (C) structures discussed in Figure 4C,E,F in the main text. RMSD values are printed for each pair of structures. The original dimer is in translucent greyscale, while the **1b/p** is blue for visibility.

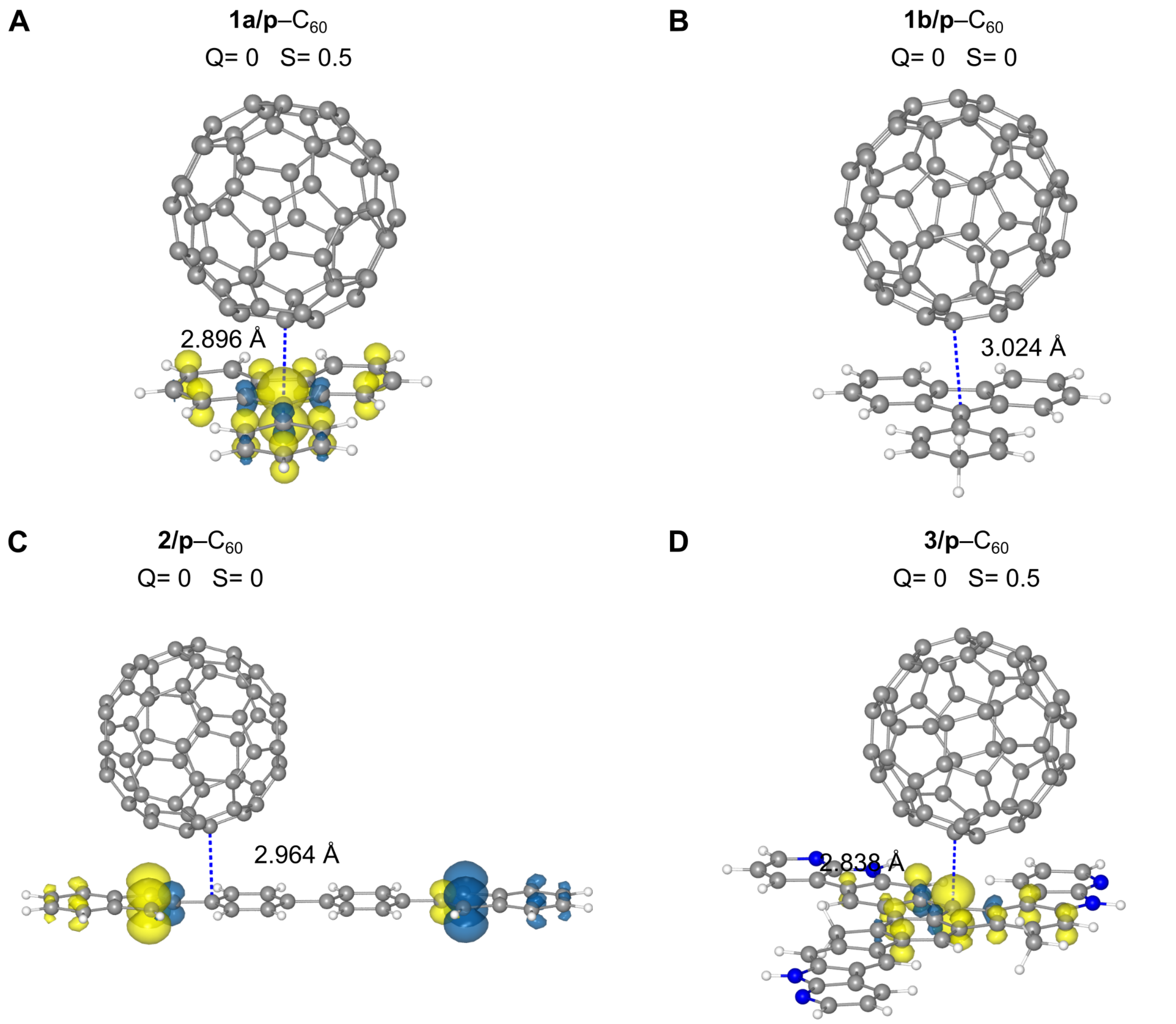

Figure S5: Structures of neutral vdW-bonded dimers (A) **1a/p**–C_{60}, (B) **1b/p**–C_{60}, (C) **2/p**–C_{60}, (D) **3/p**–C_{60} and their calculated spin densities. The shortest intermolecular C-C distances are highlighted and labeled. /**p** indicates that the energy minimization was done under constraints of planary. Yellow and light blue isosurfaces represent α and β spin densities, respectively.

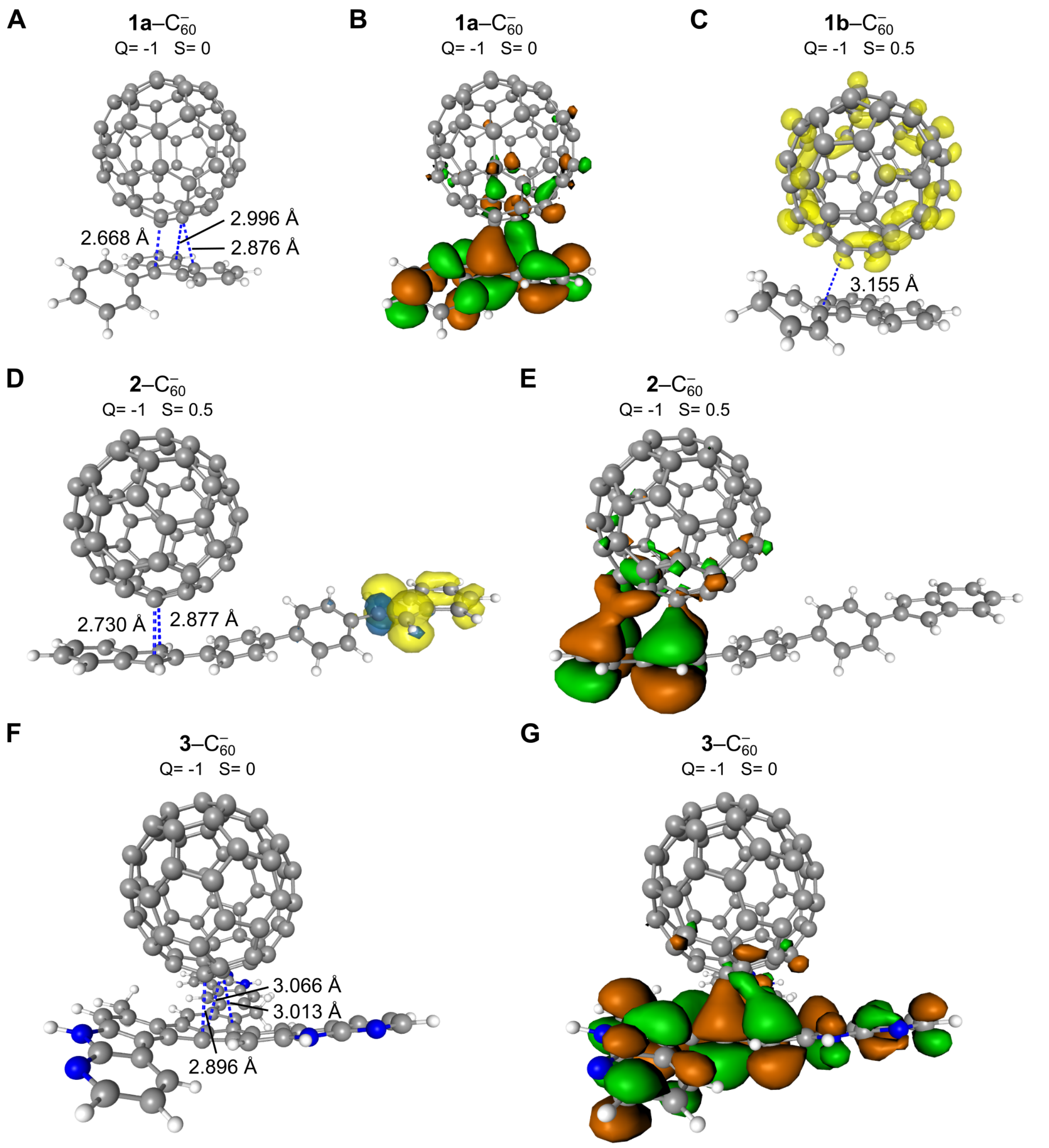

Figure S6: Unconstrained optimized structures of dimers (A, C, D, and F) of **1a**, **1b**, **2,** and **3** with C_{60}^{-}. Bonding multicenter MOs (B, E, and G) of **1a**, **2,** and **3** with C_{60}^{-}. Calculated spin density (C and D) of **1b**–C_{60}^{-} and **2**–C_{60}^{-}. Yellow and light blue isosurfaces represent α and β spin densities, respectively.

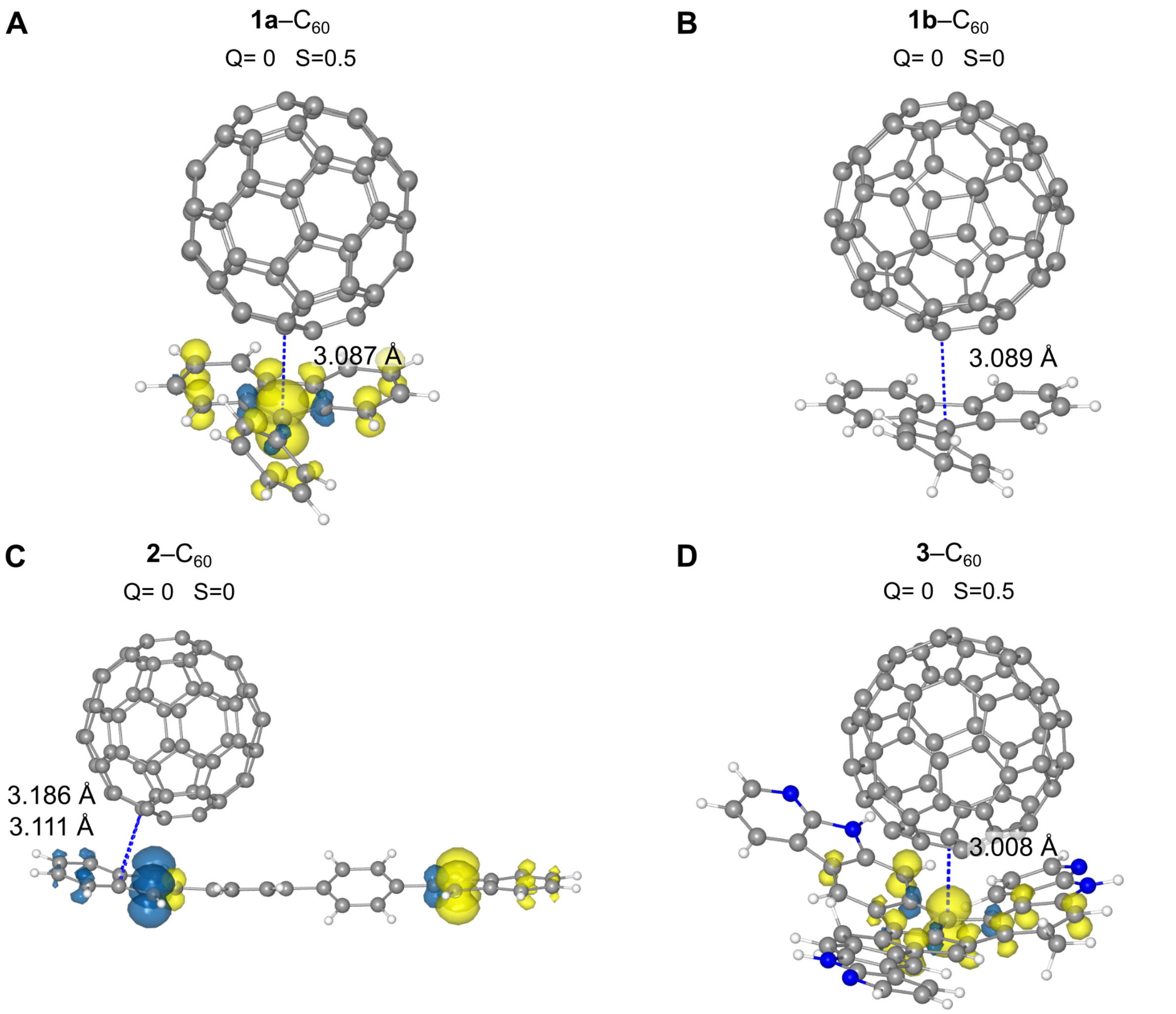

Figure S7: Unconstrained optimized geometries dimers of **1a**, **1b**, **2** and **3** with C_{60}. (A) structure and spin density of **1a**–C_{60}, (B) structure of **1b**–C_{60}, (C) structure and spin density of **2**–C_{60}, (D) structure and spin density of **3**–C_{60}. Yellow and light blue isosurfaces represent α and β spin densities, respectively.

Table S2: Interaction energies and summed atomic Mulliken charges (∑q) of gas-phase unconstrained dimers in two states, neutral and anionic. Values in brackets include ZPVE corrections. All values refer to fully optimized geometries.

M06-2X def2-TZVP	Neutral (Q = 0)			Anion (Q = -1)		
	E_{int} [kcal/mol]	$\sum q_{mol}$	$\sum q_{C_{60}^{Q}}$	E_{int} [kcal/mol]	$\sum q_{mol}$	$\sum q_{C_{60}^{Q}}$
**1a**–C_{60}^{Q}	-8.6 (-7.9)	0.048	-0.048	-12.7 (-9.2)	-0.597	-0.403
**1b**–C_{60}^{Q}	-8.6 (-8.0)	0.054	-0.054	-7.5 (-6.8)	-0.014	-0.986
**2**–C_{60}^{Q}	-7.3 (-6.5)	0.028	-0.028	-14.9 (-11.3)	-0.693	-0.307
**3**–C_{60}^{Q}	-12.0 (-11.6)	0.056	-0.056	-19.3 (-18.6)	-0.817	-0.183

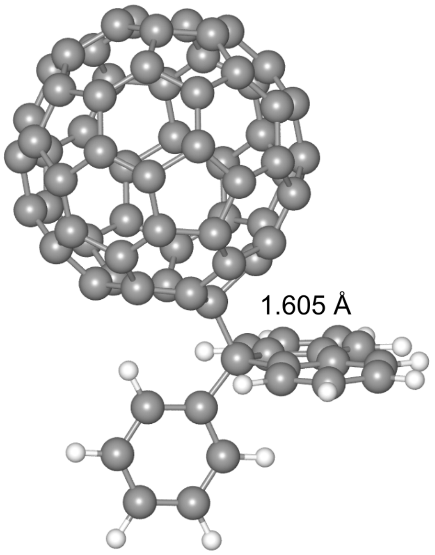

Figure S8: σ-bonded minimum structure of **1a**–C_{60}^{-} with interaction energy of -17.9 kcal/mol including ZPVE correction.

Discussion on jellium charge error and cluster calculations

The proposed experimental setup requires a formation of charged molecule attached to the AFM tip under ultra-high vacuum conditions. When performing periodic boundary condition computations with the NaCl(001) monolayer, the supercell contains a negative charge. Due to the requirement for electroneutral cell, the vacuum region is filled with homogeneous background countercharge, so called jellium. Although the charged C_{60} molecule is 17 Å apart from its image in neighboring cell, and the periodicity in z direction is 100 Å, we have observed irregular behavior in the charged supercell calculations. Namely, the interaction of C_{60}^{-} with the NaCl(001) surface at 6 Å vacuum gap (geometry from **1a**–C_{60}^{-} dimer on surface) reaches -7.8 kcal/mol. For comparison, the interaction energy of C_{60} at comparable geometry is -0.3 kcal/mol. Even at a distance of ~45 Å from the surface, C_{60}^{-} shows interaction of -4.2 kcal/mol. For this reason, we carried out cluster single-point energy calculations to verify this error. When the periodicity is removed and the NaCl slab is left without change, C_{60}^{-} interacts with the slab by -1.8 kcal/mol at the dimer height of ~6 Å and no interaction is present at ~45 Å. We carefully analysed the Mulliken atomic charges to make sure that the bonding in the cluster calculations are the same and we found no deviation from the periodic structures.

In conclusion, jellium countercharge introduced a significant error to the total energies, reaching almost 8 kcal/mol. Thanks to the insulating nature of NaCl, the nonperiodic cluster calculations yielded qualitatively eqivalent electronic structure of the system, avoiding the error introduced by jellium.

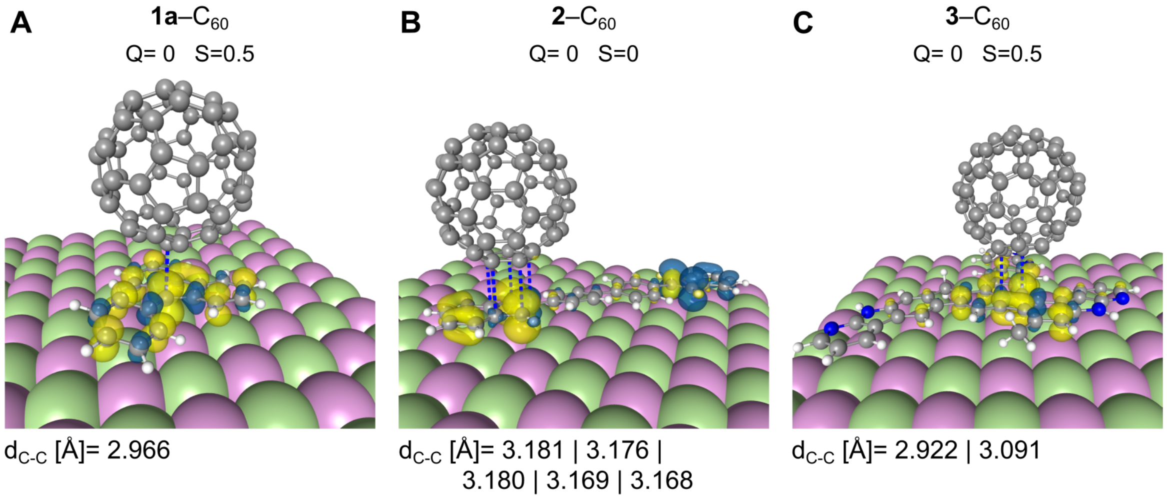

Figure S9: Neutral dimers relaxed on a fixed periodic NaCl(001) monolayer. (A) **1a**–C_{60}, (B) **2**–C_{60}, (C) **3**–C_{60}. Dashed blue lines highlight close contacts, and their values are listed below the images. Yellow and light blue isosurfaces represent α and β spin densities, respectively. Mauve and lime balls represent Na and Cl, respectively.

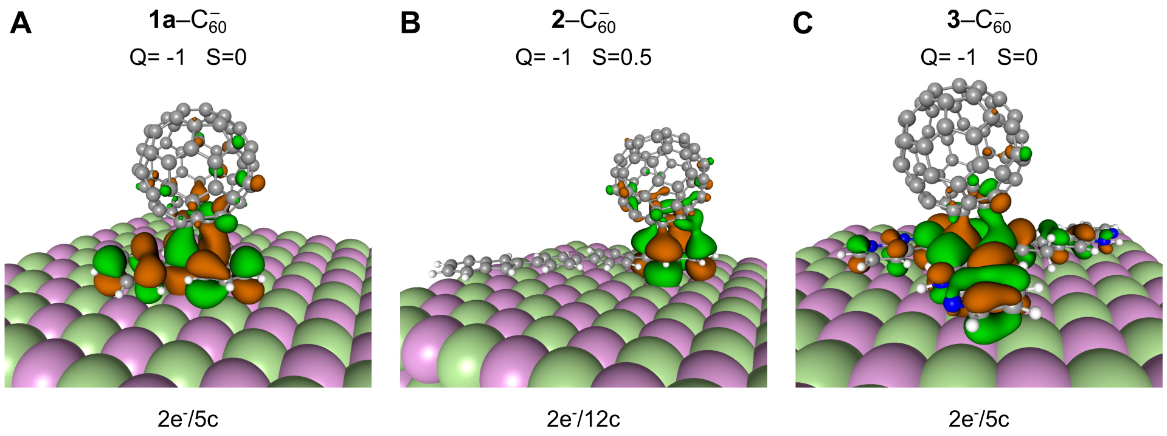

Figure S10: Bonding MOs of the anionic dimers relaxed on a fixed periodic NaCl(001) monolayer. (A) **1a**–C_{60}^{-}, (B) **2**–C_{60}^{-}, (C) **3**–C_{60}^{-}. Pancake bond characteristics are given below the structures. Mauve and lime balls represent Na and Cl, respectively.

Table S3: Interaction energies calculated for fixed dimer geometries in vacuum, with geometries obtained from energy minimization computation on a periodic NaCl(001) monolayer.

M06-2X def2-TZVP	Neutral (Q = 0)			Anion (Q = -1)		
	E_{int} [kcal/mol]	$\sum q_{mol}$	$\sum q_{C_{60}^{Q}}$	E_{int} [kcal/mol]	$\sum q_{mol}$	$\sum q_{C_{60}^{Q}}$
**1a**–C_{60}^{Q}	-7.0	0.089	-0.089	-12.5	-0.570	-0.430
**2**–C_{60}^{Q}	-4.2	0.067	-0.067	-12.9	-0.618	-0.382
**3**–C_{60}^{Q}	-5.8	0.096	-0.096	-19.9	-0.724	-0.276
